# Mechanochemical route to the synthesis of nanostructured Aluminium nitride

**DOI:** 10.1038/srep33375

**Published:** 2016-09-21

**Authors:** S. A. Rounaghi, H. Eshghi, S. Scudino, A. Vyalikh, D. E. P. Vanpoucke, W. Gruner, S. Oswald, A. R. Kiani Rashid, M. Samadi Khoshkhoo, U. Scheler, J. Eckert

**Affiliations:** 1 Department of Materials Engineering, Birjand University of Technology, Birjand, Iran; 2 Department of Chemistry, Faculty of Sciences, Ferdowsi University of Mashhad, 91775-1436 Mashhad, Iran; 3 Institute for Complex Materials, IFW Dresden, Helmholtzstraße 20, D-01069 Dresden, Germany; 4 Institut für Experimentelle Physik, TU Bergakademie Freiberg, Leipziger Str. 23, 09596 Freiberg, Germany; 5 Institute for Materials Research (IMO), Hasselt University, Wetenschapspark 1, 3590 Diepenbeek, Belgium; 6 Department of Materials Engineering, Ferdowsi University of Mashhad, 91775-1111, Mashhad, Iran; 7 Leibniz-Institut für Polymerforschung Dresden e.V., Hohe Str. 6, 01069 Dresden, Germany; 8 Erich Schmid Institute of Materials Science, Austrian Academy of Sciences, Jahnstraße 12, A-8700 Leoben, Austria; 9 Department Materials Physics, Montanuniversität Leoben, Jahnstraße 12, A-8700 Leoben, Austria

## Abstract

Hexagonal Aluminium nitride (h-AlN) is an important wide-bandgap semiconductor material which is conventionally fabricated by high temperature carbothermal reduction of alumina under toxic ammonia atmosphere. Here we report a simple, low cost and potentially scalable mechanochemical procedure for the green synthesis of nanostructured h-AlN from a powder mixture of Aluminium and melamine precursors. A combination of experimental and theoretical techniques has been employed to provide comprehensive mechanistic insights on the reactivity of melamine, solid state metal-organic interactions and the structural transformation of Al to h-AlN under non-equilibrium ball milling conditions. The results reveal that melamine is adsorbed through the amine groups on the Aluminium surface due to the long-range van der Waals forces. The high energy provided by milling leads to the deammoniation of melamine at the initial stages followed by the polymerization and formation of a carbon nitride network, by the decomposition of the amine groups and, finally, by the subsequent diffusion of nitrogen into the Aluminium structure to form h-AlN.

Among the nitrides for electronic applications, AlN is of particular interest because of the positive combination of properties, including high thermal conductivity, large band gap, low dielectric constant and low linear thermal expansion coefficient^1^,^2^. Moreover, nanostructured AlN can be used to prepare technologically important AlGaN alloys or to form nanoscale heterojunctions with other semiconductors enabling band offset engineering in nanodevices^3^.

The conventional processing route for the production of AlN consists of the carbothermal reduction of Al_2_O_3_ in a flowing N_2_/NH_3_ gas mixture at temperatures above 1673 K^1^,^4^. This process requires the use of toxic ammonia along with a long time exposure of the reactants at elevated temperatures. The first systematic attempt to reduce the reaction temperature was made by Paseuth and Shimada, who prepared a homogeneously dispersed Al_2_O_3_-amorphous carbon mixture by heating Aluminium oleic emulsion at 873 K in air^5^. Nanostructured AlN was then produced by heating the mixture at temperatures at 1423–1473 K in NH_3_. They proposed that NH_3_ first reacts with amorphous carbon to form gaseous HCN species, which are thermodynamically favoured at temperatures above 1123 K, and then these active radicals react with Al_2_O_3_ and shift the reduction-nitridation process to lower temperatures according to the following reaction:
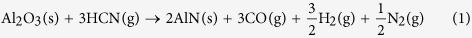


A different processing route, based on a solid state metathesis, has been proposed recently for the synthesis of nitrides using solid nitrogen-containing organic compounds (SNCOCs). In this route, metal oxide is first mixed with a SNCOC, such as cyanamide^6^, dicyanamide^7^ or melamine^8^,^9^; then the mixture is heated in sealed quartz tubes to form the corresponding nitride. The mechanism underlying this route includes the dissociation of the SNCOCs at temperature above 873 K, which releases nitrogen-containing radicals like C_2_N^+2^, C_3_N^+2^, C_3_N^+3^, C_3_N^+4^ and HCNH^+^^10^. Such active radicals first reduce the oxide into the corresponding metallic element and then the corresponding metal nitride is formed by nitridation of metallic particles with the residual carbon nitride^6^. Although, a wide variety of nitrides such as TiN, AlN, GaN and VN nanoparticles have been prepared by this approach, the relatively high temperature required for the reaction completion and the need for sealed conditions limit the extensive use of this synthesis route.

A way to overcome this problem is offered by the mechanochemical approach, which permits the room temperature synthesis of a wide spectrum of nanostructured materials^11^,^12^,^13^. Conventional high-energy ball milling is a prominent mechanochemical technique, in which the energy necessary for the reaction is provided by the mechanical force imparted by the ball impacts^13^. Most of the studies on the mechanochemical synthesis of nitrides are concentrated on the solid-gas metathesis (SGM), in which metals or metal oxides are ball milled under a gaseous N_2_ or NH_3_ atmosphere^14^,^15^. However, in the AlN synthesis by SGM full conversion of reactants into products is hardly achieved within a reasonable time due to the low reactivity of gaseous N_2_ and poor contact of the solid-gas reactants^14^,^15^. Additionally, the use of N_2_ or NH_3_ requires controlled atmosphere conditions and proper gas sensing solutions. Thus, the replacement of a gaseous with a solid nitrogen source would provide an alternative route to overcome these issues. In this context, the use of SNCOCs as a nitrogen source in solid state reaction by ball milling represents an eco-friendly processing route which benefits from the room temperature synthesis and absence of toxic gaseous reactants. This has been demonstrated recently by the mechanochemical synthesis of nanostructured AlN using SNCOCs precursors such as melamine^16^ and diaminomaleonitrile (DAMN)^17^.

In spite of the positive aspects of AlN formation via the mechanochemical route, the formation of the intermediates and by-products has not been fully understood yet. Moreover, there is no detailed description of the mechanically-induced phase evolution and formation of nitrides using SNCOCs. Accordingly, in the present work, we have performed a comprehensive investigation of the solid state synthesis of nanostructured AlN by ball milling of Al and melamine. Melamine (C_3_H_6_N_6_) is a trimer of cyanamide, with a 1,3,5-triazine skeleton, which contains 66.6 wt.% nitrogen (higher than DAMN). It is a safe, cheap and non-explosive nitrogen-rich material, which is widely used for manufacturing melamine dinnerware, laminate flooring, fire-retardants and as additive in paints and plastics^18^; therefore, it represents an excellent precursor for the synthesis of nitrides. The objective of this study is to explore the mechanochemical reaction of Al with melamine from both computational and experimental points of view, paying special attention to the solid state reaction mechanism, identification of intermediates, and structural characterization of the final nanostructured AlN product.

## XRD analysis

**Figure 1:**
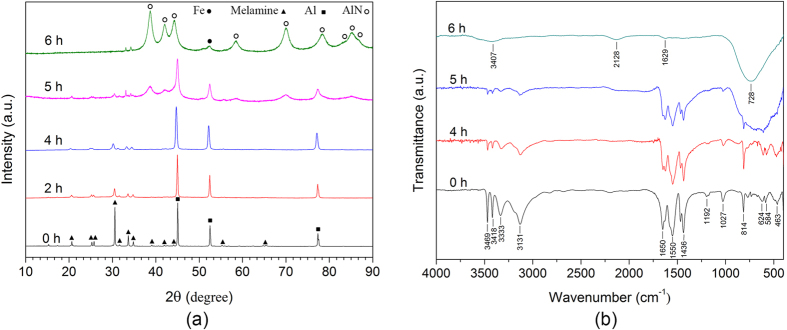
(**a**) XRD patterns and (**b**) IR spectra of the reactants before milling (0 h) and after various milling times.

The phase evolution of the reactants as a function of the milling time is shown in Fig. 1(a) along with the XRD pattern of the Al and melamine starting mixture (0 h). Milling up to 4 h leads to a significant reduction of peak intensities along with peak broadening due to the decrease of the crystallite size and to the creation of structural defects. Beside the presence of Al and melamine, no additional phases are visible at this milling stage. The formation of the h-AlN occurs after 4 h and conversion appears complete after milling for 6 h. At this stage, traces of iron due to the contamination from the milling media can be observed. The broad diffraction peaks at 6 h indicate the nanostructural character of the AlN phase. This is corroborated by the Rietveld analysis, which reveals a mean crystallite size of AlN of ~11 nm.

## FTIR analysis

Figure 1(b) displays the FTIR analysis of the materials obtained at various milling times. As Aluminium is an IR inactive solid, the spectrum obtained at 0 h is representative of the melamine chemical structure. The peaks at 3000–3500 cm^−1^ can be assigned to the stretching vibration mode of the amine groups in the melamine molecule. The bending mode of these bonds is observed in the range of 1600–1650 cm^−1^. The bands at frequencies between 1100–1600 cm^−1^ correspond to the C‒N and C = N stretching vibration modes of the triazine ring, while the signals at 450–1050 cm^−1^ correspond to the C‒NH_2_ along with the ring vibrations. The peaks at 814 cm^−1^ and 1027 cm^−1^ are associated with out-of-plane bending and breathing modes of the triazine ring, respectively. In comparison to the starting powder mixture, no significant changes are observed in the peak positions of the powder mixture after milling for 4 h. However, the intensity of the amine groups is reduced significantly indicating partial deammoniation of melamine. The elimination of the amine groups continues in the powder milled for 5 h and the intensity of the -NH_2_ bond shows further reduction in the corresponding IR spectrum. The ring vibrations at 1100–1600 cm^−1^ are unchanged and the presence of the characteristic ring band at 814 cm^−1^ implies that the triazine ring structure is retained in the intermediates. At this stage, a broad and strong peak at 600–800 cm^−1^, which can be is assigned to the Al‒N bond stretching vibration mode^19^, is observed. This confirms the XRD results (Fig. 1(a)) showing the formation of AlN at this milling time. With further milling up to 6 h, the reaction of the residual Al particles with nitrogen of the triazine rings proceeds. This can be inferred by considering the strong broad peak at 728 cm^−1^, which can be ascribed to the formation of AlN, and the disappearance of the characteristic ring bands at 1100–1600 cm^−1^ and 814 cm^−1^ (Fig. 1(b)). The weak peaks at 1629 cm^−1^ and 3407 cm^−1^ can be assigned, respectively, to the bending and stretching vibration modes of the hydroxyl group (‒OH) resulting from the moisture absorption and surface hydrolysis of nanostructured AlN. The origin of the peak at 2128 cm^−1^ cannot be explained by the current results and needs further experiments to be clarified.

## DFT simulation of the reactants interaction

In our previous work, we demonstrated that milling of pure melamine (i.e. without Al) has no effect on its structure even after prolonged milling times except for grain refinement and induced crystal defects^16^. Therefore, the partial deammoniation of melamine observed above is a clear sign of the structural destabilization related to the presence of the Aluminium particles. In order to clarify the role of Al on the chemical destabilization of the melamine molecules, a systematic computational study has been conducted using the DFT method.

**Figure 2:**
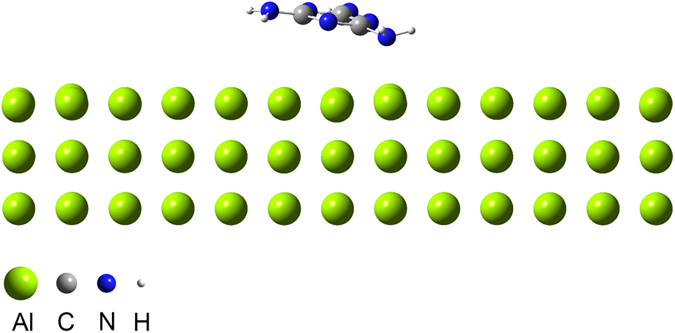


Figure 2 presents the optimized geometrical features of a melamine molecule positioned above the Al (100) plane (for additional details see Supplementary Information). The melamine molecule is oriented with its amine group directed toward the Aluminium surface. The adsorption energy (E_ads_) is given by:
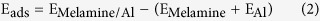
where E_Melamine/Al_ is the energy of the system with the melamine molecule adsorbed on the Al surface and E_Melamine_ and E_Al_ are the energies of the isolated melamine and Al slab, respectively. The calculated E_ads_ based on Equation (2) is −0.894 eV (−20.61 kcal/mol), indicating that such a physical adsorption is energetically favourable. In contrast, by neglecting dispersion effects E_ads_ = 0.066 eV, which implies that the largest part of the binding energy comes from Van der Waals interactions. The resulting values of bond lengths in both pure melamine and melamine after adsorption on the Al (100) surface display a large discrepancy in the C‒NH_2_ bond length directed toward the Aluminium surface. The C‒NH_2_ bond length of the adsorbed molecule is 0.04887 Å longer than the average value for the free molecule, and 0.05781 Å longer than the average of the other two bonds. The NH_2_ group bonded to the surface is tilted out of the molecular plane by about 6°, indicating that the contribution of this amine group to the ring charge balance is reduced and that the nitrogen atom of the amine group is likely to interact with the Al (100) surface. Moreover, the hydrogen atoms of the NH_2_ group are oriented in opposite directions with respect to the Al surface and their bond length is increased by 0.009738 Å.

The DFT results describe the potential interactions in the Al-melamine system without introducing any external energy or force into the system. Thus, when the system is exposed to high-energy ball milling, the amine bond directed toward the Aluminium surface can be broken, leading to the deammoniation of melamine. Indeed, this finding has been supported by the significant reduction in the intensity of the –NH_2_ bands in the IR spectra (material milled for 4 and 5 h in Fig. 1(b)).

## XPS analysis

**Figure 3:**
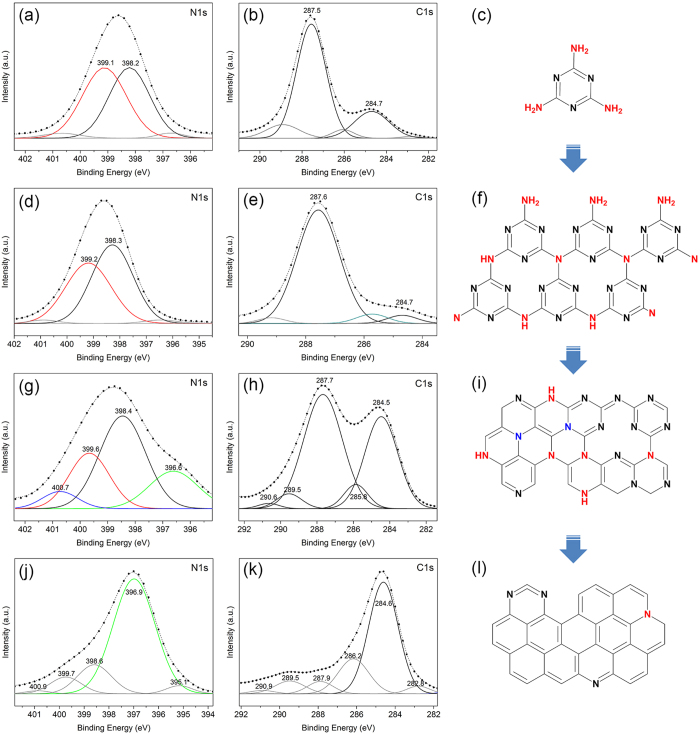
Deconvoluted XPS N1s and C1s spectra, and schematic representation of the corresponding structures for the (**a**–**c**) reactants (0 h) and samples milled for (**d**–**f**) 4 h, (**g**–**i**) 5 h and (**j**–**l**) 6 h. The spectral components are indicated by solid lines, their sum by dashed lines and the experimental spectrum by dots.

In order to identify the reaction intermediates, further structural characterization of the milled powders has been performed by XPS (Fig. 3). The deconvoluted N1s spectra reveal that the ratio of the integrated peak areas corresponding to pyridinic N (at ~398.4 eV) associated with the sp^2^ hybridized aromatic N bonded to two carbon atoms (C‒N=C) and tertiary N (at ~399.6 eV) in the form of N‒(C)_3_, H‒N‒(C)_2_ or H_2_N‒C^20^,^21^,^22^,^23^, changes with increasing the milling time. For the reactants (0 h), the equal peak areas of these two components indicate that the same ratio of pyridinic and tertiary N occurs in the melamine molecule (Fig. 3(a)). This balance is changed in the powder milled for 4 h (Fig. 3(d)): the peak area of tertiary N is reduced due to deammoniation of melamine and formation of short-range CN_x_ networks. This hypothesis is supported by the comparison of the IR vibration modes of the intermediates (Fig. 1(b)) with those of graphitic carbon nitrides described in the literature^24^,^25^. As milling proceeds to 5 h, Al starts to react with the carbon nitride network to form AlN, which is thermodynamically more favoured than other Aluminium compounds, such as AlH_3_ and Al_4_C_3_, consequently leaving carbon atoms with the aromatic ring structure in the domains (C‒C and N‒C=N bonds at 284.5 and 287.7 eV, respectively, as shown in Fig. 3(h)). This leads to the formation of highly condensed CN domains with a higher C/N ratio (Fig. 3(i)). The high condensation of the CN_x_ network is inferred from the appearance of the sp^2^ C‒C (relatively strong peak at 284.5 eV in Fig. 3(h)) and graphitic N bonded to three carbon atoms in the aromatic rings (peak at 400.7 eV in Fig. 3(g)). This is a characteristic peak in the N1s spectrum of carbon nitrides, which indicates the conjugated CN rings and the formation of a polymeric carbon nitride^21^,^22^,^26^. Nitrogen removal from the CN_x_ network continues until the Al particles are completely consumed and AlN particles (the major peak at 396.9 eV in Fig. 3(j)) are formed along with a by-product carbonaceous material with graphitic structure (dominant C‒C bond at 284.6 eV in Fig. 3(k)). A small amount of residual nitrogen is expected to be trapped in the carbonaceous structure. Considering this fact, the aforementioned undefined band at 2128 cm^−1^ in the IR spectrum of the 6 h milled sample (Fig. 1(b)) can be ascribed to the incorporation of nitrogen in the carbonaceous structure in the form of C≡N or N=C=N bonds (Fig. 3(l))^27^. Contrary to the XPS results, the formation of the carbonaceous phase has not been detected by XRD (Fig. 1(a)), probably because of the low degree of crystallinity. In Fig. 3(j), the other weak components at 398.5 eV, 399.7 eV and 400 eV can be attributed to Al‒O‒N (or C=N‒C), NH_x_ and Al‒N‒O, respectively, which have been frequently reported for nanostructured AlN due to the surface oxidation^28^,^29^.

The structural evolution of melamine during milling is schematically illustrated in Fig. 3. Melamine is polymerized during the initial stages of milling to form CN_x_ networks. When the reaction of Al with N of the CN_x_ domains starts, the pyridinic, tertiary and graphitic nitrogen atoms in the carbon nitride are substituted by the aromatic sp^2^ C‒C rings and, finally, the CN_x_ network transforms to a graphitic carbonaceous structure. It is worth to mention that the above reaction of Al with the CN_x_ network is in contrast to the results obtained by the carbothermal reduction route, which also uses SNCOCs as a nitrogen source^6^,^8^. In other words, in the conventional thermal treatment, AlN is synthesized by reaction of the oxide with gaseous active species produced due to thermal decomposition of SNCOCs and, therefore, the process must be performed under sealed conditions to avoid the loss of the reactive gaseous constituents^6^,^7^,^8^. In contrast, in the current mechanochemical procedure, nitridation occurs in the solid state between Al and N atoms of the polymerized melamine (CN_x_ networks) without need for the gas controlling equipment. Furthermore, additional experiments on milling under unsealed atmospheric conditions (i.e. with opened vial valve during milling) led to similar results with respect to the closed system with the only significant difference consisting in higher oxygen contamination.

## ^27^Al NMR analysis

**Figure 4:**
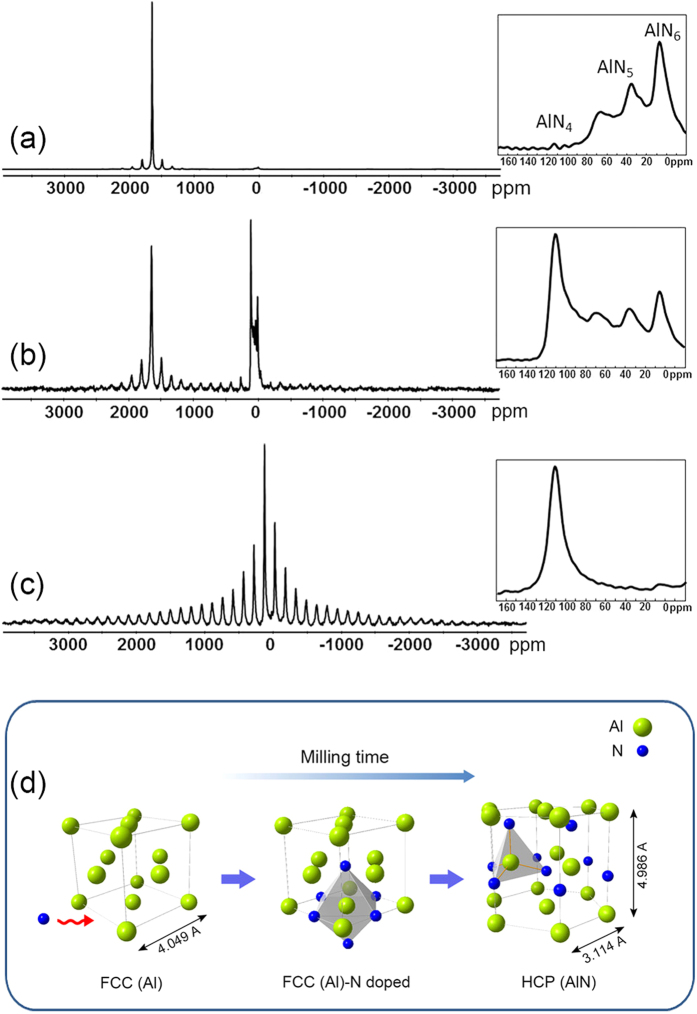
^27^Al NMR spectra of the samples after various milling times: (**a**) 4 h, (**b**) 5 h and (**c**) 6 h. Minor peaks equally distant from the central lines are from spinning side bands. The spectra are normalized to maximal intensity. (**d**) Schematic structural evolution of Al during milling.

In order to study the phase transformation from face-cantered cubic (FCC) Al to h-AlN, the evolution of the Aluminium structure during milling has been analysed by solid state ^27^Al NMR at milling intervals between 4 and 6 h (Fig. 4). The NMR spectrum of the sample milled for 4 h (Fig. 4(a)) shows a strong peak at about 1640 ppm corresponding to the characteristic Knight shift of FCC-Al^30^. This agrees with the XRD results, which indicate the presence of unreacted Al at this milling time (Fig. 1(a)). Additionally, low intensity peaks in the range 0–120 ppm (consisting of four distinct components; see inset in Fig. 4(a)) are also detected in this spectrum. The higher intensity peak at 8 ppm can be attributed to AlN_6_, indicating that N atoms mainly occupy octahedral sites around Al^31^,^32^. The other peaks at 38 and 68 ppm can be assigned to the five-coordinated Al (AlN_5_) or distorted octahedra and to a mixed form of N with oxygen impurity (AlN_x_O_y_)^31^,^33^. When the milling time reaches 5 h, the relative intensity of the peaks in the range 0–120 ppm increases significantly (Fig. 4(b)) and the signal at 114 ppm assigned to tetrahedral-coordinated Aluminium (AlN_4_)^31^,^32^,^33^ becomes dominant (inset in Fig. 4(b)). This can be ascribed to the diffusion of N into Al and to the formation of hexagonal AlN with AlN_4_ coordination. On the other hand, the relative intensity of the peak at 1640 ppm decreases due to the consumption of Al, which is no longer visible at the end of milling process (Fig. 4(c)). Therefore, the inset in Fig. 4(c) exclusively shows a single peak at 114 ppm assigned to the AlN_4_ of h-AlN. The observed strong spinning side bands in the ^27^Al spectrum of this sample result from the presence of paramagnetic contamination (i.e. iron) in the Aluminium nitride introduced during milling.

The insets in Fig. 4(a–c) suggest a possible mechanism for the structural transition from FCC-Al to h-AlN (Fig. 4(d)). Atomic N from the organic precursor diffuses into the Al structure (preferentially into the octahedral sites) at the initial stages of milling. While Al retains its FCC structure to minimize the structural mismatch, the diffusion of N continues until the Al structure becomes super-saturated. When milling exceeds 5 h, further N diffusion is accompanied by the structural rearrangement of the super-saturated Al and its conversion to hexagonal AlN. As a consequence, AlN_6_ in FCC-Al gradually transforms to AlN_4_ in h-AlN (inset in Fig. 4(b)) and finally disappears when the reaction is complete (inset in Fig. 4(c)).

## DFT simulation of N-incorporated Al structure

Although the NMR results confirm the diffusion of N into the Al structure, no (equilibrium) solid solubility is expected for the Al-N system at room temperature^34^. Such a discrepancy can be explained by the non-equilibrium character of milling, which permits solid−state alloying beyond the equilibrium solubility limit and the formation of metastable phases, such as amorphous, quasicrystalline or nanostructured materials^35^. Thus, the milling process must be analysed using sophisticated theories, such as a quantum theory, which explains the nature and behaviour of matter and energy on the atomic and subatomic levels^36^. Considering this fact, the solid solubility of N in Al and the sites preferentially occupied by N have been investigated using DFT (for details about site occupancy see Supplementary Information). A primary assumption is that the atomic nitrogen is detached from the organic precursor as a result of milling and allowed to penetrate into the Al slab (see Supplementary Information; Figure S1).

Regardless of which site is occupied, the diffusion of N into the Al slab is energetically favourable. Although for single N interstitials the tetrahedral site is the most favourable, upon the formation of complexes with more than 4 N interstitials, the complex with N interstitials at octahedral sites becomes preferred because it can retain more N atoms around the same central Al site. This scenario agrees well with the observed variation of the ^27^Al spectral profile at initial milling times (4 h), when the AlN_6_ peak appears and becomes dominating. We suggest that continuous milling provides additional energy required for diffusion of the N atoms between different interstitial sites.

## Analysis of gaseous by-products

Our results so far have provided detailed information about the solid constituents. However, for the complete investigation of the reaction, the analysis of the gaseous by-products is necessary. For this purpose, the produced gases after milling for 6 h have been analysed by connecting the milling vial to a QMS spectrometer via a connector tube (see Supplementary Information, Figure S2). The primary gas remained inside of the connector (Ar) tube before opening the vial (red spectrum in Figure S3). The produced gases inside of the vial are then determined by subtracting the two spectra recorded before and after opening the valve (*after* – *before* = blue spectrum in Figure S3). The main difference between the spectra is the peak at 2 m/z (mass-to-charge ratio) corresponding to the formation of H_2_ (Figure S3). In addition, a small amount of CH, NH and NH_2_ species, which can occur in the form of C_x_H_y_ (such as C_2_H_2_), N_x_H_y_ and C_x_H_y_N_z_ as a result of the polymerization and condensation of melamine fragments, has been identified. This is corroborated by the ammonia-like odour detected after opening the vial valve, which can thus be attributed to the presence of these species in the gas mixture. The presence of H_2_ as the dominant gaseous by-product can be explained as follows. At the initial milling times, the absorption of the melamine on the Al surface is accompanied by the increment of the N–H bond length of the corresponding amine group. Thus, it is expected that when the C–NH_2_ bond is broken, an NH_2_ radical rapidly decomposes on the Al surface to form atomic nitrogen and H_2_ as:
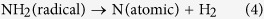


This *in situ* formed atomic nitrogen then diffuses into the Al structure and occupies the octahedral sites, whereas H_2_ is released as shown by the QMS results. The role of the reactive hydrogen may be very helpful in the process due to its protecting behaviour on the freshly broken Al metal particle surface by ball milling. Thus, this finding is in good agreement with the NMR results and DFT calculations discussed above.

## Reaction pathway

**Figure 5:**
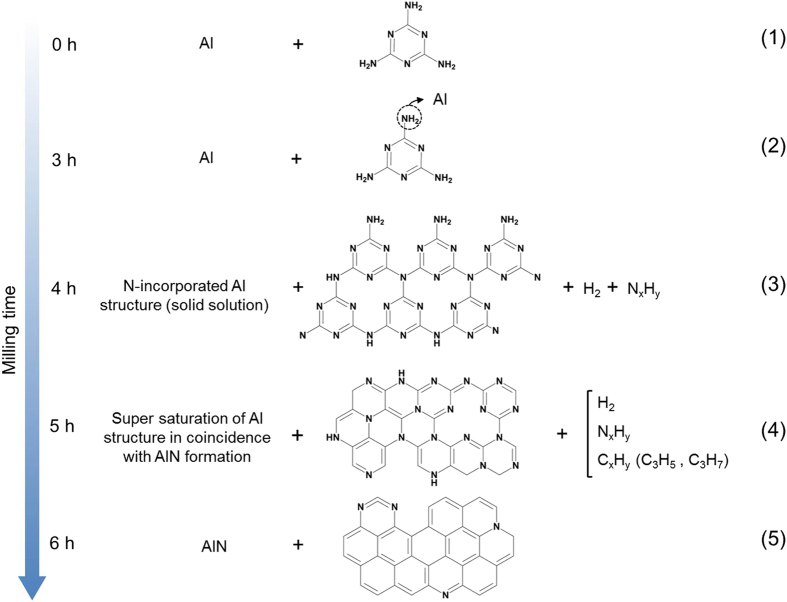


According to the results above, the overall mechanism for the mechanochemical reaction of Al and melamine can be divided into four main steps, as schematically illustrated in Fig. 5. Firstly, melamine is absorbed on the Al surface through the nitrogen of the amine group and, therefore, its contribution to the electrical charge balance of the triazine ring is reduced. In this step (3 h), the amine bond is broken and the produced NH_2_ radical is decomposed to atomic N and H_2_. Atomic N then diffuses into the Al structure and occupies the octahedral sites in the Al lattice to form an Al-N solid solution (4 h). At the same time, the melamine radicals merge to form short range CN_x_ networks. When the Al structure reaches the solid solubility limit, further diffusion is allowed by conversion of FCC-Al to h-AlN (5 h). This structural transformation facilitates the interactions of the remaining Al particles with nitrogen in the CN_x_ networks and increases the C/N ratio. This step proceeds until all Aluminium particles react with N. Finally, in the last step (6 h), a mixture of AlN and heterogeneous carbon is produced. The overall reaction can be summarized as follows:
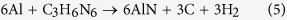


In this equation, it is assumed that the reactants are fully converted to the products and H_2_ is the only gaseous by-product. Accordingly, the weight fraction of carbonaceous component is theoretically estimated from Equation (5) to be 12.8 wt.%, which agrees well with the measured value of 9.4 wt.% obtained from combustion carbon analysis.

## SEM and HRTEM observations

**Figure 6:**
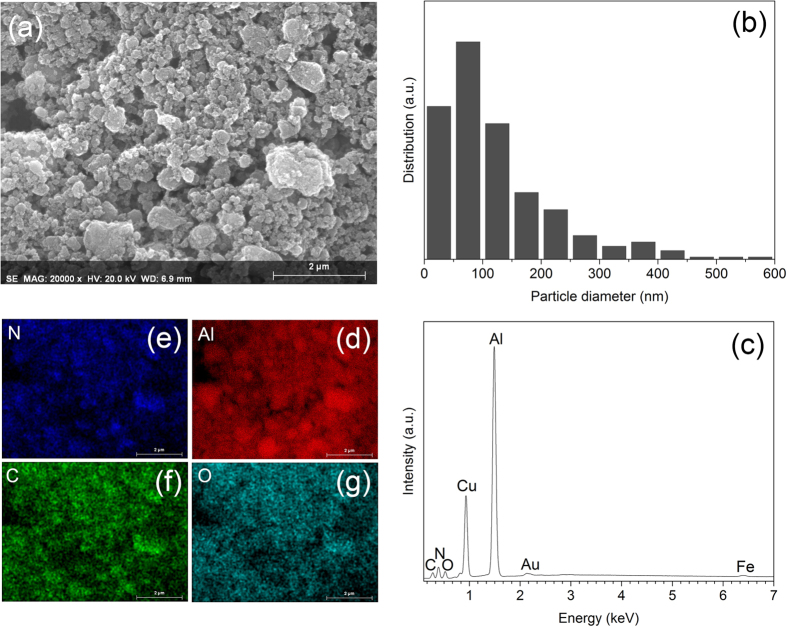
(**a**) Characteristic SEM image of the powder mixture milled for 6 h and corresponding (**b**) particle size distribution, (**c**) EDX spectrum and (**d**–**g**) EDX mapping.

SEM and HRTEM were employed to observe the morphology of the particles and to estimate their size. Figure 6(a) shows a typical SEM micrograph of the powder particles synthesized after 6 h of milling. The powder consists of aggregated particles of irregular shape, wide-range size distribution (Fig. 6(b)) and average particle size of about 150 nm. Chemical analysis of the powder shows the presence of Al, N, C, O, Cu, Au and Fe elements (Fig. 6(c)). Al and N peaks can be assigned to the formation of AlN. The C peak refers to the heterogeneous carbon produced by decomposition of the organic precursor, as already deduced from the XPS results (Fig. 3(k)). Oxygen results from the surface hydrolysis and oxidation of the powder most likely during milling as well as from the short time exposure to air during handling and SEM sample preparation. Additionally, the detection of oxygen supports the presence of AlN_x_O_y_ species identified in the NMR spectra. The origin of Cu and Au peaks is from the copper sample holder and gold coating of the powder, respectively. The Fe peak is due to iron contamination from the milling media. Figure 6(d–g) display EDX mapping of the abovementioned elements. The uniform distribution of Al, N, C and O implies that AlN is very well-mixed with the carbonaceous structure.

**Figure 7:**
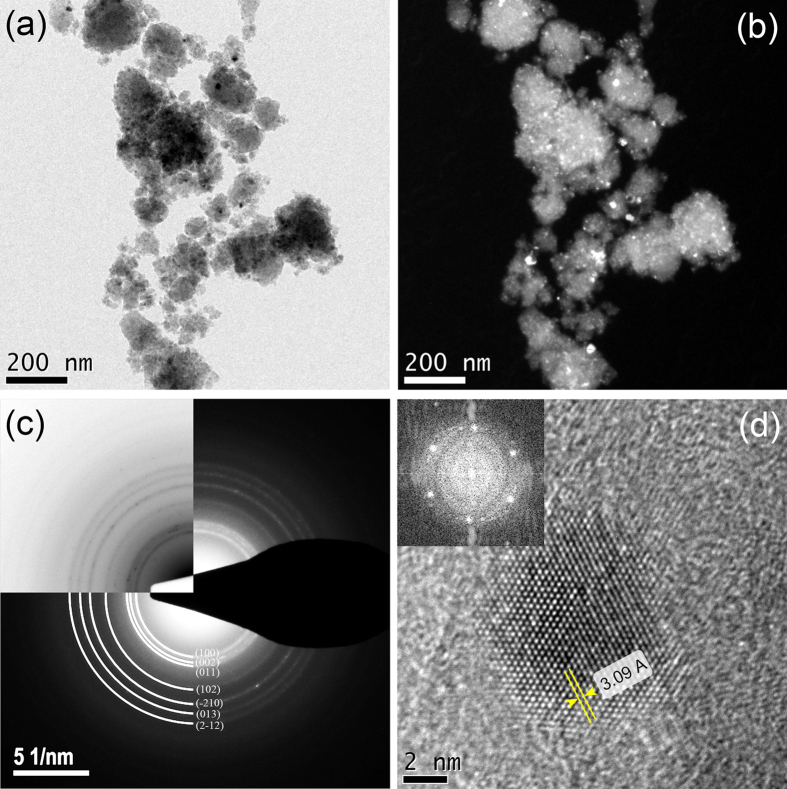
Typical (**a**) bright and dark (**b**) field TEM images of the powder mixture milled for 6 h and (**c**) corresponding SAED pattern. (**d**) FFT filtered HRTEM and inset FFT diffraction pattern of a nano-sized AlN basal plane.

Representative bright and dark field TEM micrographs of the sample milled for 6 h are shown in Fig. 7(a,b). The images confirm the formation of irregularly shaped particles with dimensions of about 100 nm. Although the crystallites of AlN and carbonaceous structure could not be distinguished in the image, the nano-metric size of the bright spots in Fig. 7(b) confirms the nanostructured nature of the constituents. The corresponding selected area electron diffraction (SAED) pattern reveals relatively sharp rings superimposed with diffuse rings (Fig. 7(c)). The sharp rings match well with the different crystallographic planes of h-AlN, whereas the diffuse rings originate from a non-crystalline phase, presumably the amorphous carbonaceous structure. Figure 7(d) shows a fast Fourier transform (FFT) filtered HRTEM micrograph of the nano-sized h-AlN basal plane. The measured interatomic distance is 3.09 Å which is consistent with the lattice parameter of h-AlN (*a* = 3.11 Å). The corresponding inset FFT diffraction pattern is indexed to prismatic planes in hexagonal AlN.

In summary, the mechanochemical synthesis of nanostructured AlN through the solid state reaction of Aluminium with melamine and the underlying reaction mechanism have been investigated and analysed in detail. The results reveal that melamine exhibits the tendency to be adsorbed on the Aluminium surface through the amine groups. Such a metal-organic interaction coupled with the high energy provided by milling leads to the deammoniation of melamine at the initial stages of milling. The resulting melamine fragments are then polymerized to form a CN_x_ network. The adsorbed amine is decomposed on the Al surface to form nitrogen and H_2_. The *in situ* formed atomic N then diffuses into the Aluminium structure and occupies first tetrahedral and octahedral interstitial sites. Upon further diffusion, the N atoms start to form complexes cantered on single Al atoms, with the preference for the octahedral interstitial site, as corroborated by ab initio calculations. When the diffusion proceeds, the FCC structure becomes super-saturated, subsequently resulting in the transformation from FCC-Al into the h-AlN structure. The reaction is completed when the remaining Al fully reacts with nitrogen from the CN networks. The final product is a fine powder mixture consisting of nanostructured AlN and non-crystalline carbonaceous material. These findings clearly indicate that the mechanochemical synthesis can be successfully employed for the fabrication of AlN using SNCOCs. This method can also be applicable to other metallic elements, such as Ti, Fe, Cr, V, and thus it may provide a simple, low cost and versatile processing route for the synthesis of a variety of nitrides of wide technological interest.

## Methods

### Sample preparation

The reactant powders, Al (Goodfellow, 99.5% purity) and melamine (Khorasan Petrochemical Co., 99.8% purity) were used as received without any further purification. The Al and melamine powders were mixed with a stoichiometric ratio of 6:1. For each milling experiment, 3 g of the powder mixture were loaded into a hardened steel vial along with the hardened steel balls (10 mm diameter) to give a ball-to-powder weight ratio of 50:1. No process control agent was used. Vial charging and any subsequent powder handling were carried out in a glove box under purified argon atmosphere (less than 1 ppm O_2_ and H_2_O). Milling experiments were performed using a Retsch PM-400 planetary ball mill at a rotating speed of 300 rpm. Milling was conducted at room temperature for various periods up to 6 h. After each milling period, the powder was completely removed from the vial.

### Materials characterization

Phase analysis was conducted by X-ray diffraction (XRD) using a Philips X’Pert X-ray diffractometer with Co Kα radiation (λ = 0.17889 nm). The Rietveld method was used to evaluate the crystallite size of the milled products. Fourier transform infrared spectroscopy (FTIR) spectra were obtained using a ThermoNicolet Avatar 370 infrared spectrometer at room temperature with KBr pellet technique. The X-ray photoelectron spectroscopy (XPS) experiments were carried out at room temperature in an ultra-high vacuum system equipped with a hemispherical electron analyzer SPECS PHOIBOS 100. Solid state ^27^Al MAS NMR spectra were obtained using a (11.7 T) Bruker Avance III 500 MHz spectrometer operating at ^27^Al resonance frequency of 130.34 MHz. ^27^Al MAS NMR spectra were acquired at a spinning frequency of 20 kHz employing a 2.5 mm MAS probe head, without proton decoupling. A single pulse of 1 μs pulse duration (<π/12 for 30 kHz rf-amplitude) was applied to ensure a quantitative excitation of the central transition. A recycle delay of 1 s and 1024 repetitions were used. ^27^Al chemical shifts were referenced to 1 M AlCl_3_ aqueous solution at 0 ppm. The spectra were fitted using Dmfit^37^. The SEM images were obtained using a Gemini 1530 (Zeiss) microscope operating at 20 kV and equipped with an energy dispersive X-ray spectrometer (EDX). High resolution transmission electron microscopy (HRTEM) measurements were carried out using a Tecnai F30 (FEI company) microscope, operating at 300 kV.

For gas analysis, the vial was equipped with a valve and milling was conducted using the same experimental conditions as discussed above. To avoid the introduction of the fine powder particles into the quadrupole mass spectrometer (QMS), which may occur due to the overpressure of the produced milling gas inside the vial, an adaptor (connector tube) was used to connect the vial indirectly to the QMS (see Supplementary Information). The tube was connected to the vial inside the glove box and the gases inside the tube have been analysed before and after opening the vial valve. The amount of solid carbon residual in the samples was determined by a combustion method using a carbon/sulphur analyser EMIA 820 (Horiba, Japan).

#### DFT calculations

To describe the interactions between Al and melamine, the long range van der Waals forces, which are essential for the calculation of adsorption energy, have to be considered in the density functional theory (DFT) calculations. However, the most widely used exchange-correction DFT functionals, such as local density approximation (LDA) and generalized gradient approximation (GGA), are governed by covalent interactions and completely neglect the dispersion effect. In order to overcome this problem, a DFT method in conjunction with semiempirical dispersion corrections, the so-called B97-D theory^38^, has been proposed and used to characterize accurately interactions in the metal – organic systems^39^,^40^. All DFT-computations are performed using the VASP code^41^. In this, the projector augmented waves (PAW) method with a kinetic energy cutoff of 500 eV is used. Exchange-correlation interactions are described using the PBE functional^42^, while the van der Waals interactions are included using the Grimme D3 semi-empirical corrections with Becke-Johnson Damping^43^,^44^. For bulk calculations a 3 × 3 × 3 cubic supercell is used, containing 108 Al atoms. A 7 × 7 × 7 Monkhorst-Pack k-point set is used to sample the first brillouin zone leading to an energy convergence within 1 meV/supercell. Calculations performed to generate a Density of States (DOS) used a more dense 11 × 11 × 11 k-point mesh. For all calculations, spin-polarization was included. Structure optimizations are performed using a conjugate gradient method and an energy convergence criterion of 10^−7 ^eV. As a result, forces on the atoms in the final structures are below 1 mV/A. To consider the possible interaction between metal and organic precursors, a melamine molecule and the Aluminium slab were first optimized separately. The melamine molecule was then positioned above the (001) facet of the Aluminium slab and geometry was again fully optimized. In order to simulate the atomic diffusion into the Al structure, four different bulk systems were considered: (*1*) pure FCC Al, which is used as reference material, (*2*) N substituting a single Al atom, (*3*) interstitial N in a tetrahedral configuration, and (*4*) interstitial N in an octahedral configuration. With 1 N atom per supercell, the N content is less than 1%. In addition, N atoms are separated by distances exceeding 12 Å, making their interaction negligible. We have extended the bulk doped systems with two complexes. In the first case, 4 N atoms are placed in the tetrahedral positions around a single Al site in such a way that the Al has a tetrahedral N surrounding (the other 4 tetrahedral sites are left empty). In the second case, 6 N atoms are placed in the 6 octahedral sites surrounding an Al site in FCC Al.

## Additional Information

**How to cite this article**: Rounaghi, S. A. *et al*. Mechanochemical route to the synthesis of nanostructured Aluminium nitride. *Sci. Rep.* **6**, 33375; doi: 10.1038/srep33375 (2016).

## Supplementary Material

Supplementary Information
