# Supplementary material for: Mechanochemical route to the synthesis of nanostructured Aluminium nitride
Source: Sci Rep. 2016 Sep 21;6:33375. doi: 10.1038/srep33375 (PMC5030656; doi:10.1038/srep33375)
Supplement: Supplementary Information [file srep33375-s1.pdf]

Supplementary Information for

Mechanochemical route to the synthesis of nanostructured Aluminium nitride

S. A. Rounaghi[1,*], H. Eshghi[2], S. Scudino[3, **], A. Vyalikh[4], D. E. P. Vanpoucke[5], W. Gruner[3], S. Oswald[3], A.-R. Kiani-Rashid[6], M. Samadi-Khoshkhoo[3], U. Scheler[7], J. Eckert[8,9]

1) Department of Materials Engineering, Birjand University of Technology, Birjand, Iran.

2) Department of Chemistry, School of Sciences, Ferdowsi University of Mashhad, 91775-1436 Mashhad, Iran.

3) Institut für Komplexe Materialien, IFW Dresden, Helmholtzstraße 20, 01069 Dresden, Germany.

4) Institut für Experimentelle Physik, TU Bergakademie Freiberg, Leipziger Str. 23, 09596 Freiberg, Germany.

5) Center for Molecular Modeling, Ghent University, Technologiepark 903, 9052 Zwijnaarde, Belgium.

6) Department of Materials Engineering, Ferdowsi University of Mashhad, 91775-1111, Mashhad, Iran.

7) Leibniz-Institut für Polymerforschung Dresden e.V., Hohe Str. 6, 01069 Dresden, Germany.

8) Erich Schmid Institute of Materials Science, Austrian Academy of Sciences, Jahnstraße 12, A-8700 Leoben, Austria

9) Department Materials Physics, Montanuniversität Leoben, Jahnstraße 12, A-8700 Leoben, Austria

*, **) Corresponding authors. rounaghi@birjandut.ac.ir, s.scudino@ifw-dresden.de

1- DFT simulation of N-incorporated Al structure

The system consisting of the Al slab and N atoms situated at different sites has been geometrically optimized. N atoms were opted to occupy the most probable positions, i.e. substitutional and interstitial sites, for which the latter includes tetrahedral and octahedral ones (Figure S1). Moreover, two bulk systems were optimized with two distinct complexes comprising of 4 and 6 N atoms respectively placed in the tetrahedral and octahedral positions around a single Al site in FCC Al.

The energy of formation has been calculated according to the following equation:

$$E_f = E_{N/Al} - (E_N + E_{Al}) \qquad (1)$$

where E_f is the energy of formation, $E_{N/Al}$ is the energy of the system when atomic N occupies an individual site in the aluminium structure (Al slab) and E_N and E_{Al} are the energies of the isolated atomic N and Al slab, respectively.

Table S1 gives the energy values calculated for the mentioned situations. The negative values of the energy indicate that the diffusion of N into Al is favourable from the thermodynamic point of view. The formation energy of the substituted N is much higher than any of the interstitial cases, indicating this site to be the least favourable of those studied. Due to the small size of the N atom, the lattice shows a minute contraction (note that we have a N concentration of 1/108, i.e. <1%). The surrounding Al atoms also move slightly toward the N atom with Al-N bond lengths of 2.77 Å compared to 2.85 for Al-Al. In contrast, N at the interstitial sites is very favourable from the energetics point of view, indicating that the N atoms are likely to diffuse from the surface into the Al bulk via the interstitial sites. In such cases, the N atom positioned in tetrahedral and octahedral sites of Al crystal clearly forms bonds with the surrounding Al atoms (bond lengths of 1.87675 Å and 2.06715 Å for tetrahedral and octahedral sites, respectively). In addition, the calculated Hirshfeld-I charges [1, 2] show that there is a clear charge transfer from the N atom to the surrounding Al, which is a typical behaviour for dopant elements [3]. The octahedral site, however, is slightly less stable than the tetrahedral one (~19 kcal/mol). Furthermore, the formation of interstitial complexes comprised of multiple N atoms appears to be very favourable, since the defect formation energy of the complexes is lower than that of a single N interstitial. Furthermore, the defect formation energy per N atom of a single complex is also more favourable than that of a single N interstitial, indicating the preference for clustering of the N interstitials. This beneficial energetics picture of the complexes allows us to consider them as seeds for the FCC-Al to h-AlN phase transition. Although the energetics of the complexes shows a preference for

clustering, the atomic structure shows that no N_2 is formed but an AlN phase is retained. This is due to the internal N-distances, which in this case are about 2.85-3 Å, therefore much longer than the N_2 bond length (~1.11 Å). Similar to the interstitially diffused single N, the defect formation energy for a single N atom in the tetrahedral complex (an Al surrounded by 4 N) is lower than in the octahedral one. The results show that the complex containing 4 N in tetrahedral sites around a single Al, is less stable than the complex containing 6 N atoms in octahedral sites around a single Al. Furthermore, constructing a complex with 8 N atoms in the tetrahedral sites around a single Al atom leads, following the structure optimization, to a structure with only 4 N in the tetrahedral sites surrounding the central Al, while the other 4 N atoms have moved to the next available tetrahedral sites which do not neighbour upon the central Al.

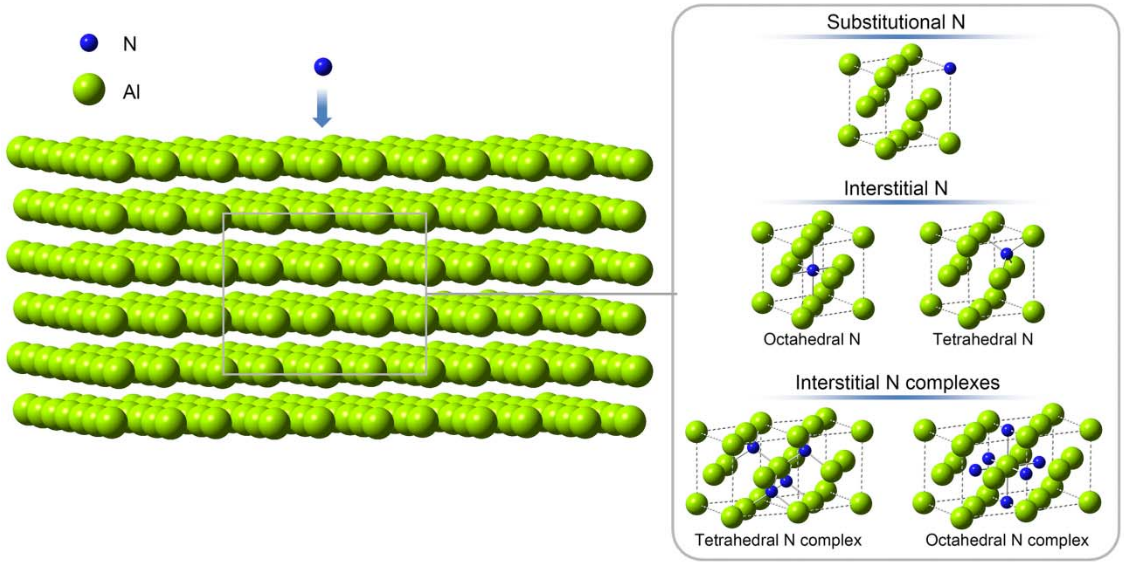

Figure S1. A schematic representation of the possible sites occupied by N atom(s) after diffusion into an Al slab.

Table S1. The lattice parameter and energies of the optimized N incorporated Al slab with doped N atoms placed at different situations.

System	$a = b = c$ (Å)	E_f (eV/cell)
Pure Al	4.040486	-
Substitutional N	4.032230	-2.1348
Tetrahedral N	4.047554	-6.0820
Octahedral N	4.043350	-5.2291
Tetrahedral N complex (4x)	4.069109	-25.896
Octahedral N complex (6x)	4.050630	-35.766

2- Analysis of gaseous by-products

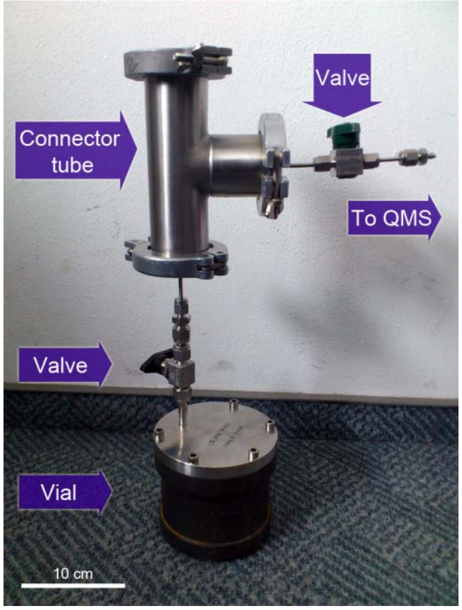

Figure S2. The vial equipped with gas analysis apparatus.

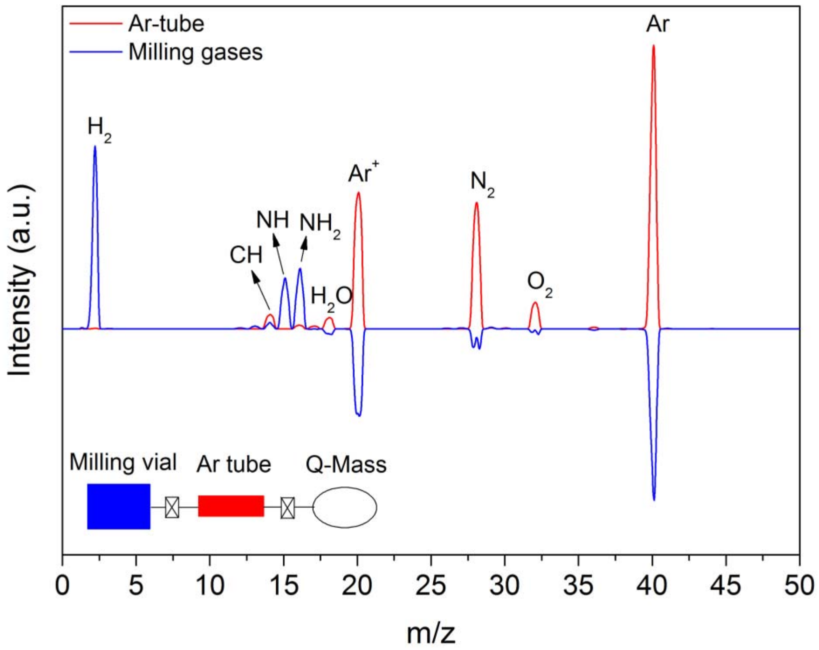

Figure S3. QMS spectra of the gaseous by-products formed after milling for 6 h and (inset) sketch of the experimental setup.

3- References

[1] D.E.P. Vanpoucke, I. Van Driessche, P. Bultinck. Reply to ‘comment on “extending Hirshfeld-I to bulk and periodic materials”, Journal of Computational Chemistry 34 (2013) 422-427.

[2] D.E.P. Vanpoucke, P. Bultinck, I. Van Driessche. Extending Hirshfeld-I to bulk and periodic materials, Journal of Computational Chemistry 34 (2013) 405-417.

[3] D.E.P. Vanpoucke, S. Cottenier, V. Van Speybroeck, I. Van Driessche, P. Bultinck. Tetravalent Doping of CeO2: The Impact of Valence Electron Character on Group IV Dopant Influence, Journal of the American Ceramic Society 97 (2014) 258-266.
